# The diagnostic accuracy of the faecal immunochemical test for the detection of early-onset colorectal cancer: an age-stratified analysis in South West England

**DOI:** 10.1038/s41416-025-03154-7

**Published:** 2025-08-22

**Authors:** Melissa Barlow, David Messenger, Ryan Preece, Amy Prowse, Gary Abel, Willie Hamilton, Samuel WD Merriel, Adam Chambers, Sarah ER Bailey

**Affiliations:** 1 University of Exeter, St Luke’s Campus, Exeter, UK; 2 Department of Coloproctology, Bristol Royal Infirmary, University Hospitals Bristol and Weston NHS Foundation Trust, Bristol, UK; 3 Colorectal Tumour Biology Group, School of Cellular and Molecular Medicine, Faculty of Life Sciences, Biomedical Sciences Building, University of Bristol, Bristol, UK; 4 Centre for Primary Care & Health Services Research, University of Manchester, Manchester, UK

**Keywords:** Diagnostic markers, Colorectal cancer

## Abstract

**Background:**

The incidence of early-onset colorectal cancer (EOCRC) is rising rapidly, with diagnoses typically occurring at a more advanced stage than late-onset CRC. In the absence of screening for younger patients, diagnosis relies on symptomatic presentation. The faecal immunochemical test (FIT) is a diagnostic triage tool for patients presenting with clinical features of CRC in primary care, though its performance in individuals under 50 years is not well established.

**Methods:**

A cohort of 38,117 symptomatic patients aged 18–49 years in upper South West England underwent FIT in primary care between 01/01/2021 and 10/07/2023. A FIT result of ≥10 µg Hb/g faeces was considered positive. In the same region, 528 EOCRC diagnoses were recorded between 01/01/2021 and 10/10/2024.

**Results:**

Of the 528 EOCRC patients, 105 (20%) underwent FIT in the year before diagnosis. The sensitivity of FIT was 92.4% (95% CI 85.5–96.7%), specificity was 88.5% (88.2–88.8%), positive predictive value (PPV) was 2.2% (1.8–2.6%), and negative predictive value was 100% (100–100%). PPVs decreased in younger age groups (18–29, 30–39, 40–49 years).

**Conclusions:**

FIT performs excellently for patients aged 40–49; however, it may not be used optimally in patients <40 years. A more targeted strategy is needed to guide investigation in younger patients.

## Background

The incidence of colorectal cancer (CRC) in patients under 50 years, generally referred to as early-onset (EO)CRC, has rapidly increased since the early 2000s [1–4]. In the UK, adults under 50 years now account for 10% of all new diagnoses, a significant rise from 5% in 2017. This concerning trend contrasts with a slight decline in overall CRC incidence in the UK over the last decade [5]. The reasons for the rise in EOCRC are unclear but are likely to have resulted from Westernisation of lifestyles typified by increased consumption of sugar, processed foods and beverages, lack of exercise, urbanisation, pollution and increased antibiotic usage that can disrupt the gut microbiome (6). EOCRC incidence exhibits a birth cohort effect, whereby the odds of developing CRC are three times higher among adults born in the mid-1980s than in the mid-1960s [6, 7].

In the absence of CRC screening for patients under 50 years, diagnosis is dependent on symptomatic presentation that frequently leads to delay because the possibility of cancer is rarely considered. Consequently, EOCRC patients are generally diagnosed at an advanced stage more often than those with late-onset CRC [8]. The faecal immunochemical test (FIT) measures the amount of haemoglobin (Hb) in a faeces sample and is a diagnostic triage tool for patients presenting with clinical features of CRC in primary care, with a threshold of ≥10 µg Hb/g of faeces used for urgent referral for CRC investigation. FIT became more ubiquitous in primary care practice after the guideline recommendation by the Association of Coloproctology of Great Britian and Ireland and the British Society of Gastroenterology (ACPPGBI/BSG) in July 2022 [9]. This prompted the UK’s National Institute for Health and Care Excellence (NICE) to update their Diagnostics Guidance 56 (DG56), incorporating a broader range of symptoms and revising the role of age in FIT recommendations [10]. This is outlined in Box 1.

While the evidence supporting the use of FIT to triage patients aged 50 years or above is well established [10–12], there is limited evidence on the performance of FIT for younger patients presenting to primary care in the UK [10]. The primary aim of this study was to determine the diagnostic performance of FIT in the detection of EOCRC, using a symptomatic population under 50 years of age in the upper South West of England.

Box 1 NICE NG12 guidelines for cancer recognition and referral: when to offer a FIT in primary care

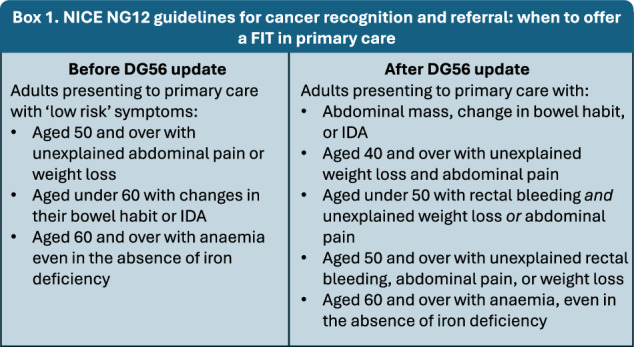

## Methods

This retrospective cohort study was conducted and reported following the Standards for Reporting of Diagnostic Accuracy Group (STARD) initiative checklist for diagnostic tests [13], as well as The Reporting of Studies Collected Using Observational Routinely-Collected Heath Data (RECORD) statement [14], an extension of Strengthening and Reporting of Observational Studies in Epidemiology (STROBE) statement [15] (Supplementary Materials 1).

### Patients and data collection

Participants eligible for inclusion in the cohort were patients aged between 18 and 49 years undergoing at least one FIT in primary care from 1^st^ January 2021 to 10th July 2023 in the Upper South West of England. We collected results from all FITs analysed by Severn Pathology in Bristol, UK, using the HM-JACKarc analyser. The sample included patients tested at any primary care practice in the Somerset, Wiltshire, Avon, and Gloucestershire (SWAG) NHS Cancer Alliance that covers the Upper South West of England. A threshold of ≥10 µg Hb/g of faeces defined a positive result. The range of possible results was <2 to >400 µg Hb/g. Patients with a result of <2 were assigned a result of 2 µg Hb/g, and patients with a result of >400 were assigned a result of 400 µg Hb/g. Data extracted from the laboratory included NHS number, patient postcode, date of birth, date of FIT, FIT result, and symptoms preceding FIT. If a patient had more than one FIT result recorded, their first test in the study period was used. Symptoms were coded as ‘low risk symptoms’ (reflecting symptoms set out in NICE guidance at the time of testing [16]), ‘change in bowel habit’, ‘non-site-specific symptoms’, ‘IDA’, or ‘two week wait pathway’ (indicating that the patient had a feature or symptom that ‘qualifies’ for an urgent suspected cancer referral). Socioeconomic status was characterised using quintiles of the area-based Index of Multiple Deprivation 2015 (IMD) [17] using postcode data. Postcodes were securely deleted after IMD assignment and after linkage to cancer diagnosis data.

All patients aged under 50 years who were diagnosed with CRC in the secondary care NHS trusts within the SWAG Cancer Alliance (Gloucestershire Hospitals NHS Foundation Trust, Royal United Hospitals Bath NHS Foundation Trust, Salisbury NHS Foundation Trust, Somerset NHS Foundation Trust, North Bristol Trust and University Hospitals Bristol and Weston NHS Foundation Trust) from 1st January 2021 to the 10th October 2024 were identified. This included patients with and without a pre-diagnosis FIT. This time frame was chosen to ensure at least 12 months follow-up from the last date of FIT sampling. (10/07/2023). Previous work suggests that a 12-month time frame is appropriate to FIT negative CRC patients who may be diagnosed through other routes, such as emergency presentations, while limiting the identification of CRC to patients diagnosed after 12 months when their CRC may not have been causing symptoms at the time of their FIT [11]. Data extracted from secondary care included NHS number, ICD-10 codes, and cancer stage at diagnosis. Proximal tumours were defined as ICD-10 codes C180 to C186 (caecum to descending colon), while codes C187, C19, and C20 were classified as distal (sigmoid colon to rectum). Early stage was defined as stage I or II, and advanced stage as stage III or IV.

The list of patients with a FIT was linked to the list of CRC diagnoses using NHS number. CRC diagnoses were retained if they occurred within 12 months of a patients’ first FIT and patients were excluded if they had a CRC diagnosis prior to their first or only recorded FIT. After linkage had been performed, an encrypted and anonymous patient identifier was assigned to each patient and all NHS numbers were securely deleted.

### Statistical analysis

Analyses were conducted using Stata SE version 18.0 [18], undertaking a complete case analysis approach. Summary statistics described all patients with a FIT, all patients with a diagnosis of CRC, and the subgroup of patients with a pre-diagnosis FIT who were subsequently diagnosed with CRC. The Chi-squared test was used to evaluate differences in proportions and the Mann–Whitney U test to assessed differences in median values of the summary statistics. Diagnostic sensitivity, specificity, positive predictive value (PPV), and negative predictive value (NPV) were estimated using Stata’s *diagt* command [19] at the following thresholds: 10, 20, 30, 40, 50, 75, and 100 µg Hb/g of faeces. This was completed for all patients, then stratified by age group: 18–29 years, 30–39 years, and 40–49 years. Due to the low number of CRC diagnoses in the 18 to 29 age group, we also included a combined age group of 18–39 years. The *ROCtab* command estimated the area under the receiver operating characteristic curve (AUC) for quantitative f-HB against CRC diagnosis [20], and the smallest sum of squares of 1-sensitivity and 1-specificity was selected to estimate the FIT threshold to maximise both sensitivity and specificity using Stata’s *rocmic* command [21].

Multivariable fractional polynomial regression estimated CRC risk by FIT result as a continuous variable to identify the threshold above which estimated CRC risk is 3% or higher, as described previously [11]. Here, the analysis was also stratified by age group (18–29 years, 30–39 years and 40–49 years). Due to the large number of patients with a FIT result >400 µg Hb/g, a post-hoc sensitivity analysis excluded patients with a FIT result of >400 µg Hb/g.

### Data governance

This project evaluated service delivery rather than changing routine clinical practice; therefore, ethical committee approval was not required. Data sharing agreements and Caldicott guardian approvals were in place between all involved parties, allowing data sharing. The use of individual NHS numbers in this study aligns with the criteria outlined in section 6 of the General Data Protection Regulation: Guidance on Lawful Processing. The processing of data is based upon GDPR Article 6(1)(e)—‘exercise of official authority’ and article 9(2)(h) ‘management of health and care services’. The legal foundation for this processing is the NHS Act 2006, Section 13E, which mandates NHS England to ensure continuous service quality improvement. This same legal basis applied to the secondary care providers contributing data.

### Patient and public involvement and engagement

Our Patient and Public Involvement and Engagement representative and co-author’s experience and reflections of being an EOCRC survivor have shaped the interpretation of results and directed areas for future research to improve the use of FIT in the younger population.

## Results

**Fig. 1:**
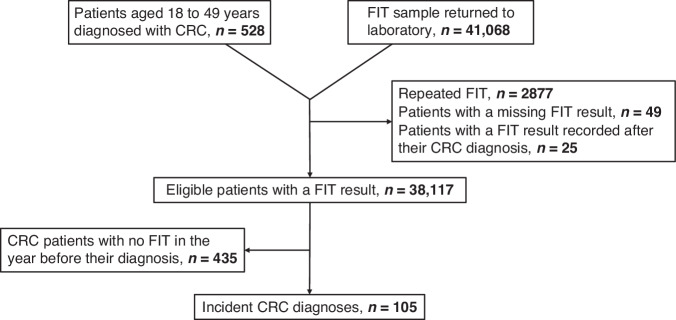
Cohort derivation. Flow diagram showing patients tested, patients diagnosed with CRC, and patients eligible for inclusion.

A total of 41,068 FIT samples were analysed by Severn Pathology between 1st January 2021 and 10th July 2023 in patients aged 18–49 (Fig. 1). Of these, 2877 were repeat FITs for the same patient, 49 results were missing, and 25 results were recorded after a CRC diagnosis, leaving a total of 38,117 eligible patients with a FIT result (the FIT cohort). Between 1st January 2021 and 10th October 2024, 528 patients under 50 years were diagnosed with CRC in the Upper South West (the CRC cohort). Approximately one-fifth of these patients (105 patients, 19.8%) had a record of a primary care-ordered FIT in the year before their diagnosis (CRC patients who underwent a pre-diagnostic FIT).

The cohort characteristics of the CRC cohort, FIT cohort, and cohort of CRC patients with a pre-diagnostic FIT are outlined in Table 1. There was little difference in age, ethnicity, and socioeconomic status across the cohorts, although there was a higher proportion of women who underwent FIT compared to the proportion of women diagnosed with CRC (62% vs 46%, p < 0.001).

**Table 1:** Cohort characteristics of patients diagnosed with CRC (CRC cohort), patients with a FIT result (FIT cohort), and CRC patients who had a FIT result in the year before their diagnosis.

	CRC cohort	FIT cohort	CRC patients with a pre-diagnostic FIT
Number of patients	528	38,117	105
Median age (IQR)	43 (39–46)	41 (34–46)	42 (40–45)
18–29 years, *n* (%)	19 (4%)	4,780 (13%)	3 (3%)
30–39 years, *n* (%)	117 (22%)	11,693 (31%)	18 (17%)
40–49 years, *n* (%)	392 (74%)	21,644 (57%)	84 (80%)
Sex	50% male	38% male	54% male
	50% female	62% female	46% female
Ethnicity	96% White	90% White	93% White
	2% Asian	4% Asian	2% Asian
	1% Black	2% Black	2% Black
Deprivation quintile, *n* (%)			
1 (most deprived)	12%	11%	13%
2	16%	15%	16%
3	20%	22%	17%
4	23%	25%	18%
5 (least deprived)	29%	26%	34%

### CRC characteristics

Two-thirds of the CRC cohort with a recorded stage at diagnosis were diagnosed at an advanced stage (266 cases, 68.7%), though stage was not recorded in 151 cases (28.1%). Among CRC patients with a determinable tumour location based on their ICD-10 code, most were diagnosed with proximal tumours (77.1%), while 22.9% were distal tumours. However, 114 CRC patients had an ambiguous ICD-10 code (C18—malignant neoplasm of colon and C189—malignant neoplasm of colon unspecified), preventing determination of the tumour location. The distribution of missing tumour location was similar between the CRC cohorts with and without a pre-diagnostic FIT. There was no difference in advanced-stage diagnoses between patients diagnosed with distal and proximal tumours.

### FIT results

**Fig. 2:**
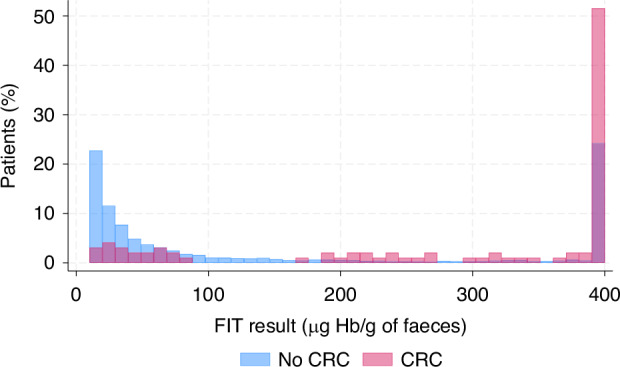
Histogram of positive FIT results, by CRC status. Red bars represent CRC patients, blue bars represent patients without CRC.

Of the 38,117 patients with a FIT test, 4474 (11.7%) had a positive result (Supplementary Material 2). A positive FIT was more common in men (12.7%) than women (11.1%) (*p* < 0.001), with a negligible difference in the median age of patients with a positive result (40 years vs 41 years, *p* < 0.001). The distribution of positive FIT results by CRC status is displayed in Fig. 2. Half of the CRC patients with a positive FIT had the maximum FIT result of ≥400 µg Hb/g (46, 47%), compared to one-quarter of the patients without CRC (1033, 24%).

### Symptoms recorded before FIT

Symptoms preceding FIT included ‘low risk symptoms’ (15,703, 56.4%), ‘change in bowel habit’ (5326, 19.1%), ‘non-site-specific symptoms’ (2485, 8.9%), ‘IDA’ (2330, 8.4%), and ‘two week wait pathway’ (1987, 7.1%). No symptom was recorded for 10,286 (27.0%) patients.

Patients who were subsequently diagnosed with CRC had a higher proportion of a specific symptom record compared to those who were not diagnosed, including change in bowel habit (27.5% vs 19.1%, *p* = 0.03) and IDA (13.8% vs 8.7%, *p* = 0.04). Additionally, a lower proportion of EOCRC patients reported low-risk symptoms (38.8% vs 56.5%, *p* = 0.001). Patients aged <40 years were more likely to have a non-specific ‘low risk symptoms’ record compared to those aged ≥40 years (71.4% vs 45.0%, *p* < 0.0001), whereas patients aged ≥40 years were more likely to have a specific symptom recorded than those aged <40 years, including: 28.6% vs 6.8% for change in bowel habit (28.6% vs 6.8%, *p* < 0.0001) and IDA (10% vs 6%, *p* < 0.0001).

### The diagnostic performance of FIT

At the ≥10 µg Hb/g of faeces threshold, the PPV of a positive test for adults under 50 years was 2.2% (95% CI 1.8–2.6%), the NPV was 100% (100–100%), the sensitivity was 92.4% (85.5–96.7%), and the specificity was 88.5% (88.2–88.8%). Stratification by age group (18–29 years, 30–39 years, and 40–49 years) indicated varying diagnostic performance by age, with improved performance with increasing age (Table 2). FIT performance was excellent for patients aged 40 to 49 years, with a PPV of 3.2% (95% CI 2.5–4.0%), NPV 100% (99.9–100%), sensitivity 92.9% (85.1–97.3%), and specificity 89.0% (88.6–89.4%). Although sensitivity, specificity and NPVs were similarly high in younger patients, the PPVs were lower: 0.4% (0.1–1.3%) for 18–29 years and 1.2% (0.7–1.9%) for 30–39 years. There was little difference in the sensitivity, specificity, NPV and AUC of FIT between the sexes or by tumour location; however, a positive FIT was slightly more predictive of CRC for males compared to females, and for distal tumours compared to proximal tumours.

**Table 2:** Diagnostic performance of FIT at the ≥10 µg Hb/g of faeces threshold for patients by age group (18–29 years, 30–39 years, and 40–49 years), sex, and tumour location (proximal and distal).

	Sensitivity, % (95% CI)	Specificity, % (95% CI)	PPV, % (95% CI)	NPV, % (95% CI)
Age group				
18 to 29	100 (29.2–100)	86.1 (85.1–87.0)	0.4 (0.1–1.3)	100 (99.9–100)
30 to 39	88.9 (65.3–98.6)	88.5 (87.9–89.1)	1.2 (0.7–1.9)	100 (99.9–100)
40 to 49	92.9 (85.1–97.3)	89.0 (88.6–89.4)	3.2 (2.5–4.0)	100 (99.9–100)
Sex				
Males	93.0 (83.0–98.1)	87.6 (87.0–88.1)	2.9 (2.2–3.7)	100 (99.9–100)
Females	91.7 (80.0–97.7)	89.1 (88.6–89.4)	1.7 (1.2–2.2)	100.0 (100–100)
Tumour location				
Proximal	95.8 (78.9–99.9)	88.3 (88.0–88.6)	0.5 (0.3–0.8)	100 (100–100)
Distal	90.0 (79.5–96.2)	88.4 (88.1–88.7)	1.2 (0.9–1.6)	100 (100–100)

**Fig. 3:**
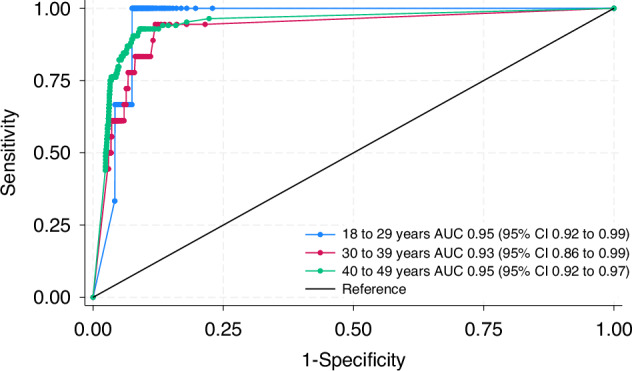
The area under the receiver characteristic curve for the diagnostic performance of FIT. The blue line represents patients aged 18–29 years, the red line represents patients aged 30–39 years, and the green line represents patients aged 40–49 years. AUC, area under the receiving operator characteristic curve.

The AUC for FIT was excellent for all three age groups: 0.95 (95% CI 0.92–0.99) for 18–29 years, 0.93 (0.86–0.99) for 30–39 years, and 0.95 (0.92–0.97) for 40–49 years (Fig. 3). The estimated FIT threshold to maximise both sensitivity and specificity was 65 µg Hb/g (95% CI 0–341 µg Hb/g), 9 µg Hb/g (0–63 µg Hb/g), 17 µg Hb/g (6–28 µg Hb/g) for the three age groups, respectively.

Table 3 outlines the diagnostic performance of FIT at thresholds of 10, 20, 30, 40, 50, 75, and 100 μg Hb/g for patients aged 18–29 years and 30–39 years, as well as a post-hoc analysis of patients aged 18–39 years due to the low number of cancer diagnoses in the 18–29 year group. The NPVs remain extremely high at all thresholds and age groups (lowest NPV value was 99.9%).

**Table 3:** The diagnostic performance of FIT at 10, 20, 30, 40, 50, 75, and 100 μg Hb/g for patients aged 18–29 years, 30–39 years, and 18–39 years.

**18–29 years**				
**Threshold, μg Hb/g**	**Sensitivity, % (95% CI)**	**Specificity, % (95% CI)**	**PPV, % (95% CI)**	**NPV, % (95% CI)**
10	100 (29.2–100)	86.1 (85.1–87.0)	0.4 (0.1–1.3)	100 (99.9–100)
20	100 (29.2– 100)	88.9 (88.0– 89.8)	0.6 (0.1–1.6)	100 (99.9–100)
30	100 (29.2–100)	90.3 (89.4–91.1)	0.6 (0.1–1.9)	100 (99.9–100)
40	100 (29.2–100)	91.3 (90.4–92.1)	0.7 (0.1–2.1)	100 (99.9–100)
50	100 (29.2–100)	91.8 (91.0–92.5)	0.8 (0.2–2.2)	100 (99.9–100)
75	66.7 (9.4–99.2)	92.8 (92.1–93.6)	0.6 (0.1–2.1)	100 (99.9–100)
100	66.7 (9.4–99.2)	93.4 (92.7–94.1)	0.6 (0.1–2.3)	100 (99.9–100)
**30–39 years**				
10	88.9 (65.3–98.6)	88.5 (87.9–89.1)	1.2 (0.7–1.9)	100 (99.9–100)
20	83.3 (58.6–96.4)	91.1 (90.5–91.6)	1.4 (0.8–2.3)	100 (99.9–100)
30	77.8 (52.4–93.6)	92.4 (91.9–92.9)	1.6 (0.9–2.6)	100 (99.9–100)
40	77.2 (46.5–90.3)	93.3 (92.8–93.7)	1.6 (0.9–2.8)	100 (99.9–100)
50	66.7 (41.0–86.7)	93.8 (93.3–94.2)	1.6 (0.8–2.8)	99.9 (99.9–100)
75	61.1 (35.7–82.7)	94.8 (94.4–95.2)	1.8 (0.9–3.1)	99.9 (99.9–100)
100	61.1 (35.7–82.7)	95.3 (94.9–95.7)	2.0 (1.0–3.5)	99.9 (99.9–100)
**18– 39 years**				
**Threshold, μg Hb/g**	**Sensitivity**	**Specificity**	**PPV**	**NPV**
10	90.5 (69.6–98.8)	87.8 (87.3–88.3)	0.9 (0.6–1.5)	100 (99.9–100)
20	85.7 (63.7–97.0)	90.4 (90.0–90.9)	1.1 (0.7–1.8)	100 (99.9–100)
30	81.0 (58.1–94.6)	91.8 (91.4–92.2)	1.2 (0.7–2.0)	100 (99.9–100)
40	76.2 (52.8–91.8)	92.7 (92.3–93.1)	1.3 (0.8–2.1)	100 (99.9–100)
50	71.4 (47.8–88.7)	93.2 (92.8–93.6)	1.3 (0.7–2.2)	100 (99.9–100)
75	61.9 (38.4–81.9)	94.2 (93.8–94.6)	1.3 (0.7–2.3)	99.9 (99.9–100)
100	61.9 (38.4–81.9)	94.8 (94.4–95.1)	1.5 (0.8–2.5)	99.9 (99.9–100)

**Fig. 4:**
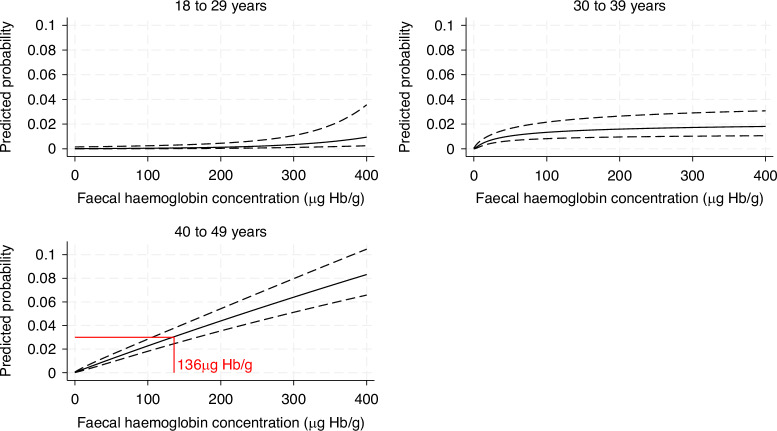
The predicted probability a patient will be diagnosed with CRC at a set f-Hb level. Results from the multivariable fractional polynomial regression model. The solid line represents the predicted probability, whereas the dashed lines represent the 95% confidence intervals.

The predicted probability a CRC diagnosis at a given faecal Hb concentration is shown in Fig. 4. Patients under 40 did not reach a 3% probability at any concentration, whereas a patient aged 40–49 years with a FIT result of 136 μg Hb/g (95% CI: 108–171 μg Hb/g) was estimated to have a 3% probability of CRC. In the sensitivity analysis, excluding patients with a FIT result >400 μg Hb/g, this was slightly lower: a FIT result of 104 μg Hb/g (95% CI: 79–138 μg Hb/g) corresponded to a 3% probability of CRC. However, for patients under 40, the probability of CRC still did not reach 3% at any concentration.

## Discussion

The diagnostic performance of FIT for EOCRC was high for all patients aged 18–49 years in this audit of routinely collected health data. However, due to the relatively low incidence of EOCRC, the PPV only exceeded 3% (the threshold at which NICE recommend urgent referral for suspected cancer) in patients aged 40–49 years at a threshold of 10 μg Hb/g. Although the PPVs were lower than 3% in the 18–39-year age groups at all thresholds up to 100 μg Hb/g, the otherwise promising diagnostic performance suggests FIT may be useful in this age range as well, possibly in combination with other factors.

Two previous studies have evaluated the performance of FIT in symptomatic patients under 50 years in the UK. D’Souza and colleagues (2021) conducted an NHS-commissioned evaluation of the diagnostic accuracy of FIT in 1103 patients referred for urgent colonoscopy between October 2017 and December 2019 [22], and Tibbs and Benton (2024) evaluated the use of FIT in 3119 symptomatic patients in primary care from June 2019 to October 2020 [23]. Both studies identified only 16 and 12 EOCRC cases, respectively, resulting in wider confidence intervals and limiting further age-stratified analysis. Despite the small number of EOCRC cases, both studies reported diagnostic sensitivity, specificity, AUC, and NPV values comparable to those in the present study, reinforcing the reliability of these findings. The PPV reported by D’Souza et al. (6.8%, 95% CI 3.7–11.4%) was approximately triple that was observed here (2.2% (2.0–2.3%), likely reflecting a higher-risk population already referred for colonoscopy, rather than patients who received a FIT as part of diagnostic work-up in primary care. The PPV reported by Tibbs and Benton was only slightly higher than what was reported here (2.7% (2.2–3.0%), potentially due their study period preceding the ACPGBI/BSG guideline recommendations.

The diagnostic sensitivity, specificity, and NPV of FIT reported here were remarkably similar to those reported in patients aged 50 and above. Bailey et al. (2021) [11] reported a sensitivity of 86.3% (95% CI 71.4–93.0%), specificity of 85.0% (83.8–86.1%), and NPV of 99.8% (99.5–99.9%) at a threshold of 10 μg Hb/g. Likewise, Withrow and colleagues (2022) [12] reported a sensitivity of 92.1% (86.4–95.5%), specificity of 91.5% (91.1– 91.9%), and NPV of 99.9% (99.9–100%) at the same threshold. The corresponding estimates in the present study were 92.4% (85.5–96.7%), 88.5% (88.2–88.8%), and 100% (100–100%), respectively. The notable difference was in the PPVs: ranging from 0.4% (0.1–1.3%) for 18–29 years, 1.2% (0.7–1.9%) for 30–39 years, 3.2 (2.5–4.0%) for 40–49 years, to 7.0% (5.1–9.3%) [11] and 8.4% (7.1–9.9%) [12] for patients aged 50 and over, reflecting the increasing prevalence of CRC with age.

The lower PPVs for the under-40s suggest FIT may be used too broadly in this age group. Indeed, the proportion of patients reporting ‘low-risk symptoms’ was significantly higher for patients under 40 years (71.4% vs 45.0%, *p* < 0.0001), which contrasts with NICE guidance in place during the study period. Incidentally, undiagnosed EOCRC patients were less likely to have reported ‘low-risk symptoms’. Under the guidance preceding the D56 update, FIT was only recommended for patients with changes in bowel habit or IDA in this age group [16]. This deviation from national guidance may reflect increased regional awareness of EOCRC in the South West, where incidence is rising fastest in the UK [6]. When ACPGBI/BSG guidelines were implemented in the region, primary care clinicians in the South West were encouraged to be permissive in their use of FIT in adults under 50 [24]. Data from Severn Pathology laboratory indicates ~20% of all FITs are performed in patients under 50 years [Correspondence 1]. Despite this, the majority of EOCRC patients (~80%) did not undergo FIT in primary care in the year before their diagnosis. Combined with the low PPVs, this suggests that FIT may not be optimally utilised in younger patients, highlighting the need to better define the risk profile of younger patients in whom FIT could enhance early detection. However, the low proportion of EOCRC patients who underwent a FIT suggests these findings may not fully represent the broader EOCRC population.

This study was a retrospective analysis of clinical data from primary care settings where FIT is currently in use, and therefore minimised spectrum bias and provided accurate findings to reflect FITs true clinical performance. All secondary care providers in the upper South West region were recruited to ensure complete case follow-up, and dedicated cancer managers ensured accurate data recording. The 12-month follow-up period allowed detection of EOCRCs that may not have been offered further investigation following a negative FIT result. However, there is a potential bias in undercounting CRC diagnoses among patients who were 49 years old at the time of their FIT but turned 50 before diagnosis. This could lead to either an underestimation of false negatives or an overestimation of false positives. Nonetheless, the number of such cases is likely very small and would not impact the overall conclusion that FIT performs excellently for patients aged 40 and over. Many of the FIT samples included in this study were likely to have been outside the contemporary guideline recommendations, although the evidence we present here, that FIT has reasonable performance in younger age groups, probably means that the off-recommendation use of FIT did not matter.

The cohorts included in this audit are largely representative of patients aged under 50 years in England; however, there is a lower representation of non-White patients and those in the most deprived IMD quintile in the South West [25]. Future research may wish to validate these findings in other regions of the UK or in countries with similar healthcare systems. As with previous reports, FIT usage was more common in women [9, 23, 26], whereas EOCRC was equally common between men and women. This disparity likely contributed to the lower PPVs observed for women, which may be explained by the higher prevalence of IDA in women [27] as well as their greater likelihood of attending primary care consultations [28].

This study supports the use of FIT to guide referral decisions in primary care for symptomatic patients aged 40 years and over at the current threshold of 10 µg Hb/g faeces. The lower PPVs observed in patients aged under 40 years suggest that applying a 20 or even 30 µg Hb/g for patients <40 years may lessen the diagnostic burden on secondary care, with only a negligible increase in false negative (missed) tests, an approach supported by previous work [23, 29]. Meanwhile, further research is needed to evaluate how symptomatic profiles impact FIT’s predictive performance in patients under 40. Given the wide variability in the predictive value of gastrointestinal symptoms for CRC [30, 31] and the lower absolute risks for each symptom [32], incorporating additional clinical factors, such as blood work, could enhance FIT’s utility in this population. Understanding FIT’s predictive accuracy in younger adults will be valuable in defining optimal FIT thresholds should the screening age be reduced below 50 years. Additionally, a composite endpoint of combining high-risk polyps and CRC may improve PPVs, albeit at the expense of NPV, but could offer a clinically meaningful strategy for earlier cancer detection.

## Conclusion

FIT performs excellently for symptomatic patients aged 40–49 years in primary care at the ≥10 µg Hb/g of faeces threshold. The lower PPVs in the 18–39-year group suggest that FIT may be being used too widely in low-risk patients in this age group, and that a different strategy could be needed to guide definitive investigation. Further research is needed on how specific symptoms and the incorporation of clinical features (such as blood work) may increase FIT’s predictive ability in younger adults. Meanwhile, a higher threshold for f-Hb may be more appropriate for patients aged under 40 years with low-risk symptoms in primary care.

## Supplementary information

RECORD statement

Patient characteristics: normal vs high FIT result

## Data Availability

Due to the sensitive nature of the research, supporting data is not available. However, programming code is available upon reasonable request.

## Code availability

Due to the sensitive nature of the research, supporting data is not available. However, programming code is available upon reasonable request.

## References

[1] Akimoto N, Ugai T, Zhong R, Hamada T, Fujiyoshi K, Giannakis M, et al. Rising incidence of early-onset colorectal cancer - a call to action. Nat Rev Clin Oncol. 2021;18:230–43. 33219329 10.1038/s41571-020-00445-1 PMC7994182

[2] Vuik FE, Nieuwenburg SA, Bardou M, Lansdorp-Vogelaar I, Dinis-Ribeiro M, Bento MJ, et al. Increasing incidence of colorectal cancer in young adults in Europe over the last 25 years. Gut. 2019;68:1820–6. 31097539 10.1136/gutjnl-2018-317592 PMC6839794

[3] Siegel RL, Miller KD, Jemal A. Cancer statistics, 2018. CA Cancer J Clin. 2018;68:7–30. 29313949 10.3322/caac.21442

[4] Exarchakou A, Donaldson LJ, Girardi F, Coleman MP. Colorectal cancer incidence among young adults in England: trends by anatomical sub-site and deprivation. PLoS ONE. 2019;14:e0225547. 31805076 10.1371/journal.pone.0225547 PMC6894790

[5] Cancer Research UK. Bowel cancer incidence statistics. Accessed 21 Oct 2024 https://www.cancerresearchuk.org/health-professional/cancer-statistics/statistics-by-cancer-type/bowel-cancer/incidence#heading-Two.

[6] Chambers AC, Dixon SW, White P, Williams AC, Thomas MG, Messenger DE. Demographic trends in the incidence of young-onset colorectal cancer: a population-based study. Br J Surg. 2020;107:595–605. 32149386 10.1002/bjs.11486 PMC7155067

[7] Siegel RL, Fedewa SA, Anderson WF, Miller KD, Ma J, Rosenberg PS, et al. Colorectal cancer incidence patterns in the United States. 1974–2013. JNCI J Natl Cancer Inst. 2017. 10.1093/jnci/djw322. 10.1093/jnci/djw322 PMC6059239 28376186

[8] The Lancet Gastroenterol Hepatology Addressing the rise of early-onset colorectal cancer. Lancet Gastroenterol Hepatol. 2022;7:197 35093209 10.1016/S2468-1253(22)00003-6

[9] Monahan KJ, Davies MM, Abulafi M, Banerjea A, Nicholson BD, Arasaradnam R, et al. Faecal immunochemical testing (FIT) in patients with signs or symptoms of suspected colorectal cancer (CRC): a joint guideline from the Association of Coloproctology of Great Britain and Ireland (ACPGBI) and the British Society of Gastroenterology (BSG). Gut. 2022;71:1939–62. 35820780 10.1136/gutjnl-2022-327985 PMC9484376

[10] National Institute for Health and Care Excellence. Quantitative faecal immunochemical testing to guide colorectal cancer pathway referral in primary care [DG56]. 2023. https://www.nice.org.uk/guidance/dg56.

[11] Bailey SER, Abel GA, Atkins A, Byford R, Davies S-J, Mays J, et al. Diagnostic performance of a faecal immunochemical test for patients with low-risk symptoms of colorectal cancer in primary care: an evaluation in the South West of England. Br J Cancer. 2021;124:1231–6. 33462361 10.1038/s41416-020-01221-9 PMC8007716

[12] Withrow DR, Shine B, Oke J, Tamm A, James T, Morris E, et al. Combining faecal immunochemical testing with blood test results for colorectal cancer risk stratification: a consecutive cohort of 16,604 patients presenting to primary care. BMC Med. 2022;20:116. 35287679 10.1186/s12916-022-02272-w PMC8920746

[13] Bossuyt PM, Reitsma JB, Bruns DE, Gatsonis CA, Glasziou PP, Irwig L, et al. STARD 2015: an updated list of essential items for reporting diagnostic accuracy studies. BMJ. 2015;351:h5527. 26511519 10.1136/bmj.h5527 PMC4623764

[14] Benchimol EI, Smeeth L, Guttmann A, Harron K, Moher D, Petersen I, et al. The REporting of Studies Conducted using Observational Routinely-collected Health Data (RECORD) Statement. PLoS Med. 2015;12:e1001885. 26440803 10.1371/journal.pmed.1001885 PMC4595218

[15] von Elm E, Altman DG, Egger M, Pocock SJ, Gøtzsche PC, Vandenbroucke JP. The Strengthening the Reporting of Observational Studies in Epidemiology (STROBE) statement: guidelines for reporting observational studies. Lancet. 2007;370:1453–7. 18064739 10.1016/S0140-6736(07)61602-X

[16] National Institute for Health and Care Excellence. Suspected cancer: recognition and referral (NICE Guideline, No. 12). 2015. https://www.nice.org.uk/guidance/NG12. 32040285

[17] Department for Communities and Local Government. The English Index of Multiple Deprivation (IMD) 2015-Guidance. 2015. https://www.gov.uk/government/statistics/english-indices-of-deprivation-2015.

[18] StataCorp. Stata statistical software: release 18.5. 2025. https://www.stata.com/stata18/.

[19] Seed P. DIAGT: Stata module to report summary statistics for diagnostic tests compared to true disease status. Stat Softw Component 2001.

[20] StataCorp LLC. roctab - Nonparametric ROC analysis. 2008. https://www.stata.com/manuals/rroctab.pdf.

[21] Froud R, Abel G. Using ROC curves to choose minimally important change thresholds when sensitivity and specificity are valued equally: the forgotten lesson of Pythagoras. Theoretical considerations and an example application of change in health status. PLoS ONE. 2014;9:e114468. 25474472 10.1371/journal.pone.0114468 PMC4256421

[22] D’Souza N, Monahan K, Benton SC, Wilde L, Abulafi M. Finding the needle in the haystack: the diagnostic accuracy of the faecal immunochemical test for colorectal cancer in younger symptomatic patients. Colorectal Dis. 2021;23:2539–49. 34240526 10.1111/codi.15786

[23] Tibbs RE, Benton SC. A service evaluation of the use of faecal immunochemical tests in symptomatic patients aged under 50 years presenting to primary care. Ann Clin Biochem Int J Lab Med. 2024;61:48–54. 10.1177/00045632231189386 37414413

[24] Personal Communication with the Somerset, Wiltshire, Avon, and Gloucestersire FIT Implementation Group, 2025.

[25] Down L, Barlow M, Bailey SER, Mounce LTA, Merriel SWD, Watson J, et al. Anaemia, ethnicity and cancer incidence: a retrospective cohort study in primary care. Br J General Pract. 2025;0762. 10.3399/BJGP.2024.0762 PMC12754673 40174989

[26] van Melle M, Yep Manzano SIS, Wilson H, Hamilton W, Walter FM, Bailey SER. Faecal immunochemical test to triage patients with abdominal symptoms for suspected colorectal cancer in primary care: review of international use and guidelines. Fam Pract. 2020;37:606–15. 32377668 10.1093/fampra/cmaa043 PMC7571772

[27] Dugan C, MacLean B, Cabolis K, Abeysiri S, Khong A, Sajic M, et al. The misogyny of iron deficiency. Anaesthesia. 2021;76:56–62. 33682094 10.1111/anae.15432

[28] Wang Y, Hunt K, Nazareth I, Freemantle N, Petersen I. Do men consult less than women? An analysis of routinely collected UK general practice data. BMJ Open. 2013;3:e003320. 23959757 10.1136/bmjopen-2013-003320 PMC3753483

[29] Pin‐Vieito N, García Nimo L, Bujanda L, Román Alonso B, Gutierrez‐Stampa MÁ, Aguilar‐Gama V, et al. Optimal diagnostic accuracy of quantitative faecal immunochemical test positivity thresholds for colorectal cancer detection in primary health care: a community‐based cohort study. United Eur Gastroenterol J. 2021;9:256–67. 10.1177/2050640620949714 PMC8259257 32778002

[30] Herbert A, Rafiq M, Pham TM, Renzi C, Abel GA, Price S, et al. Predictive values for different cancers and inflammatory bowel disease of 6 common abdominal symptoms among more than 1.9 million primary care patients in the UK: a cohort study. PLoS Med. 2021;18:e1003708. 34339405 10.1371/journal.pmed.1003708 PMC8367005

[31] Astin M, Griffin T, Neal RD, Rose P, Hamilton W. The diagnostic value of symptoms for colorectal cancer in primary care: a systematic review. Br J Gen Pract. 2011;61:e231–43. 21619747 10.3399/bjgp11X572427 PMC3080228

[32] Stapley SA, Rubin GP, Alsina D, Shephard EA, Rutter MD, Hamilton WT. Clinical features of bowel disease in patients aged <50 years in primary care: a large case-control study. Br J Gen Pract. 2017;67:e336–e344. 28347985 10.3399/bjgp17X690425 PMC5409433

