# Supplementary material for: The diagnostic accuracy of the faecal immunochemical test for the detection of early-onset colorectal cancer: an age-stratified analysis in South West England
Source: Br J Cancer. 2025 Aug 22;133(8):1170–7. doi: 10.1038/s41416-025-03154-7 (PMC12533231; doi:10.1038/s41416-025-03154-7)
Supplement: Supplementary file 2 — Patient characteristics: normal vs high FIT result [file 41416_2025_3154_MOESM2_ESM.docx]

**Supplementary Materials 2. Patient demographics by FIT result.**

Normal FIT result <10μg Hb / g. Positive FIT result ≥10μg Hb / g.

|  | **Normal FIT result** | **Positive FIT result** |
| --- | --- | --- |
| Number of patients, n (%) | 33,643 (88%) | 4,474 (12%) |
| CRC diagnoses | 8 (8%) | 97 (92%) |
| Median age (IQR)  18-29 years, n (%)  30-39 years, n (%)  40-49 years, n (%) | 41 (34 – 46)  4,112 (12%)  10,333 (31%)  19,198 (57%) | 40 (34 – 45)  668 (15%)  1,360 (30%)  2,446 (55%) |
| Male sex, n (%)  Female sex, n (%) | 12,613 (87%)  21,024 (89%) | 1,844 (13%)  2,628 (11%) |
| Ethnicity | 90% White  4% Asian  2% Black | 92% White  3% Asian  1% Black |
| Deprivation quintile, n (%)  1 (most deprived)  2  3  4  5 (least deprived) | 11%  15%  22%  25%  26% | 12%  16%  22%  26%  24% |
